# Impaired Cardiorespiratory Fitness and Muscle Strength in Children with Normal-Weight Obesity

**DOI:** 10.3390/ijerph17249198

**Published:** 2020-12-09

**Authors:** Martin Musálek, Cain C. T. Clark, Jakub Kokštejn, Šarka Vokounova, Jan Hnízdil, Filip Mess

**Affiliations:** 1 Faculty of Physical Education and Sport, Charles University, José Martího 31 Praha 6 Veleslavín, 162 52 Prague, Czech Republic; kokstejn@ftvs.cuni.cz (J.K.); vokounova@ftvs.cuni.cz (Š.V.); 2 Faculty of Health and Life Sciences, Coventry University, Richard Crossman Building, Jordan Well, Coventry CV1 5RW, UK; cain.clark@coventry.ac.uk; 3 Pedagogical Faculty, Jan Evangelista Purkyně University, Pasteurova Street 1, 400 96 Ústí nad Labem, Czech Republic; Jan.Hnizdil@ujep.cz; 4 Department of Sport and Health Sciences, Technical University of Munich, Georg-Brauchle-Ring 60/62, 80992 Munich, Germany

**Keywords:** normal-weight obesity, cardiorespiratory, children, muscle strength, physical fitness

## Abstract

Despite the health-related implications of normal-weight obesity in children, very little research has explored the fundamental associations between this status and important long-term health parameters. Therefore, the aim of the current study was to investigate the physical fitness of children with normal-weight obesity, in comparison to normal-weight non obese and overweight and obese counterparts. A total of 328 middle-school-aged children (9.8 ± 0.5 y) took part in this study (*n* = 44 normal-weight obese; *n* = 237; normal-weight non obese; *n* = 47 overweight and obese). Height, weight, and body-fatness were measured. Four physical fitness tests were conducted: (1) Multistage fitness test; (2) shuttle run 4 × 10 m; (3) sit-ups for 60 s; (4) the broad jump. Welch’s analysis of variance (ANOVA), stratified by sex, with post-hoc testing where necessary, was performed. Children with normal-weight obesity had significantly (*p* < 0.01) lower cardio-respiratory and muscular fitness than normal-weight non obese peers. In addition, normal-weight obese and overweight and obese boys had comparable deficits in strength and explosiveness of lower limbs, speed coordination, and endurance, compared to normal-weight non obese counterparts. Normal-weight obese children appear to have similar deficits in PF as their overweight and obese peers, compared to normal-weight non obese counterparts, whilst boys had larger deficits than girls.

## 1. Introduction

Physical fitness (PF) developed throughout childhood is well recognised as an important health parameter [1], where low levels can lead to an increased incidence of metabolic and cardiovascular risk factors, and coronary artery disease, respectively [2]. Stodden et al. [3], conceptualised the relationship between health, physical fitness, physical (in)activity, and motor competencies, therein describing developmental mechanisms influencing physical activity trajectories of children. In this model, physical inactivity is connected with low levels of physical fitness and low levels of motor competence (effectively solving motor tasks), resulting in a higher risk of obesity and its negative health consequences.

Previous research has repeatedly demonstrated the existence of a significant link between PF and obesity in children. Children who are obese (OB), with a high level of body fat concomitant to a high body mass index (BMI), were found to have a significantly lower PF level compared to normal weight non-obese peers, and at a higher risk of metabolic or cardiovascular diseases [4]. However, although normal-weight obesity is shown to have serious health consequences [5], very little is known about its association with motor development. Normal-weight obesity is defined by an excessive amount of total body fat, concomitant with average or normal BMI values, where individuals with normal-weight obesity have a higher prevalence of metabolic syndrome, pro-inflammatory status or atherosclerosis, similar to OB peers [6]. Excess body fat, along with normal BMI, indicates that normal-weight obese individuals likely have decreased lean mass development compared to normal weight non-obese peers. In a child population, this assumption was supported by Musalek et al. [7], where normal-weight obese pre-pubertal school children had a significantly lower lean mass area in the extremities, which are used for manipulation (upper extremities) or transportation (lower extremities).

Indeed, substantial evidence indicates that an association exists between physical fitness and health status in children and adolescents [8]. In Ortega et al. [9], the authors concluded that both cardio-respiratory and muscular fitness are related with cardiovascular disease risk factors, and that muscular fitness and speed/agility appear capable of eliciting a positive effect on bone health. Moreover, an important extension of the aforementioned associations is that physical fitness level is also positively linked to long term health status. Therefore, it is conceivable that children with greater PF in childhood present a lower risk of health-related diseases (cardiovascular, metabolic) in adulthood [10]. In addition, it has been found that PF is strongly associated with adherence physical activity regimen [11] and that physical activity in childhood can track into adulthood, with consequential health implications [12]. Such information conceivably corroborates the finding of Musalek et al. [13], where normal-weight obese pre-school children had significantly poorer fundamental motor skills (FMS); where balance and postural stability, as tenets of FMS, were shown to be highly correlated with the development of motor coordination. Moreover, sex-related differences appear to be manifest in children regarding FMS, highlighting the additional importance of considering sex, across ages [13].

However, to the author’s knowledge, despite some contemporary literature regarding the implications of normal-weight obesity, there has been no study that has investigated the associations between normal-weight obesity and PF in an elementary-school aged population. Further, the difference in PF between normal-weight obese children and their overweight and obese peers remains wholly misunderstood, as does the interaction of sex. Indeed, such information is essential for identifying the seriousness of normal-weight obesity, in boys and girls, in relation to PF, which can significantly influence long-term physical, social, and mental development. In addition, better understanding may be beneficial to key stakeholders in the education sector, particularly given the extensive period of time children spend in school, concomitant to the numerous national child testing and surveillance programmes already undertaken; however, cross-sectional work must initially address the gap in the literature.

Therefore, the aim of the current cross-sectional study was to investigate the physical fitness of children with normal-weight obesity, in comparison to normal-weight non obese and overweight and obese counterparts, with consideration of sex-related differences.

## 2. Materials and Methods

The current study was conducted using a cross-sectional research design, where participants were selected from a specific demographic region of the capital of the Czech Republic. The methods and results of current study were described in line with recommendation of *The Strengthening the Reporting of Observational Studies in Epidemiology (STROBE) Statement: guidelines for reporting observational studies* [14].

### 2.1. Participants and Study Design

Participants were recruited as part of the University Research projects Social-Sciences Aspects of Human Movement Studies I and Social-Sciences Aspects of Human Movement Studies (PRVOUK P39 and PROGRES Q19) conducted at Charles University, Faculty of Physical Education and Sport. Details of sampling procedure and eligibility criteria are provided below in sections (a) Selection of schools and (b) Selection of participants

#### 2.1.1. Selection of Age Category

We focused on school children aged 9–11 y, because, in children, this represents a stable period in motor development [15]. We included only participants aged from 9.00 to 10.99 years. In the Czech Republic, this age range corresponds to 3rd and 4th grades of school.

#### 2.1.2. Selection of Schools

**Figure 1:**
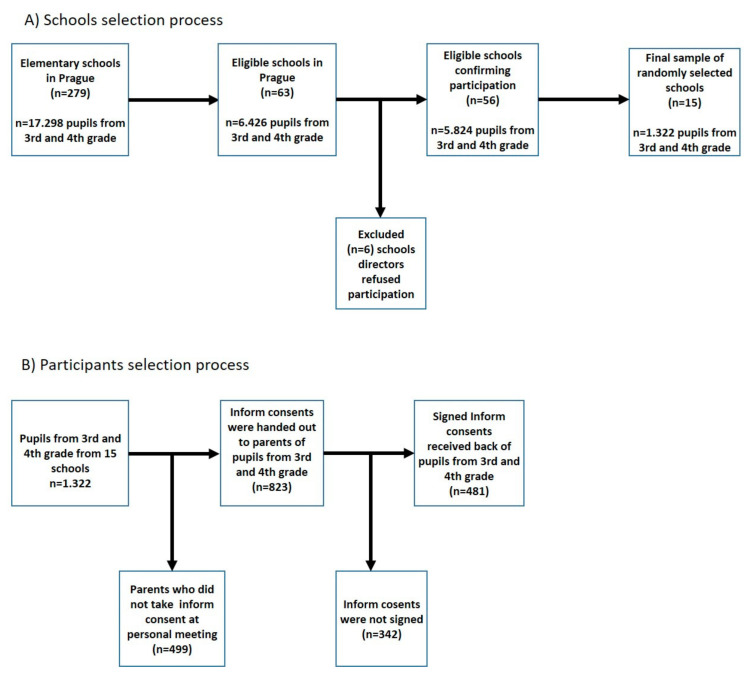
Schools and participant selection flow charts.

Elementary schools from Prague, the capital city of the Czech Republic, were selected. In the first step we defined 5 regions of Prague to cover schools located in the centre as well as in the suburbs in which we wanted to collect data. Regions were defined as range of distance from the centre of Prague: 1st region centre radius 0–2 km from centre; 2nd region radius 2–4 km; 3rd radius 4–8 km; 4th region radius 8–12 km; 5th region suburb radius 12–16 km. Then, we defined the number of schools to be chosen from each region considering the east–west and south–north orientations. Eligibility criteria for school sample selection were: (1) public school; (2) no language, technical, art, or sport specialization; (3) no special requirements (e.g., involving children with mental, neurological or physical diagnoses); (4) proportional size of classes (*n* = 22–28 pupils) in the selected age category. Based on these criteria we obtained the list of 63 eligible elementary schools from the Ministry of Education of the Czech Republic. The number of eligible schools differs considering Prague regions: 1st = 8 schools; 2nd = 11 schools; 3rd = 13 schools; 4th = 14 schools; 5th = 18 schools. Fifty-six of these schools (88.9%) agreed to participate in this study (Figure 1). Based on a randomization procedure carried out in the open-source Randomizer software (Dublin, Ireland) (www.random.org), fifteen non-specialized elementary schools were selected (two schools from region 1 and two schools from region 2, three schools from region 3 and three schools region 4, and five schools from region 5). 

#### 2.1.3. Selection of Participants in Schools

In each school, a meeting with the school management and parents/guardians was conducted during regular school meetings. We presented the purpose of the study and informed them that the procedures involved in our study are in accordance with the ethical standards of the responsible Czech national committee on human experimentation and with the Helsinki Declaration of 1975, revised in 2000. Further, we declared that the research was approved by the Ethics Committee of the Faculty of Physical Education and Sport, Charles University No.169/2015 and No. 144/2017. Written informed consent forms were handed out to parents/guardians, and only those children from whom we received written informed consent signed by them and by their parents were involved in the study.

Out of 823 informed consent forms that were handed out, a total of 481 (58.4%) were signed by parents and children (Figure 1). In total, *n* = 481 children were recruited and the data were immediately anonymized, as each child received a unique code.

In the 481 children, we measured selected anthropometry parameters and physical fitness. However, the final sample of children used in current study had to be selected based on the criteria that are described in detail in Section 2.3.

Data were collected in the period from October 2017 to March 2018 and the data collection was carried out in all schools at the same time of the day, between 8:00 a.m. and 11:40 a.m. Prior to measurements being taken, teachers and parents were instructed to have children dressed in light clothing, e.g., t-shirt, shorts, and sport shoes.

### 2.2. Instruments

#### 2.2.1. Anthropometry

All anthropometric measurements were conducted according to the “Anthropometric Standardization Reference Manual” produced by Lohman et al. [16].

In each school, anthropometry measurement was carried out with the same four academically trained examines. Further, in each school three separate rooms were reserved where body height, body weight and skinfold measurement were measured. These rooms passed all requirements given by Czech hygiene law 410/2005 Sb for education of children. In each room, an appointed teacher was present during measurement.

Body height was measured using a portable anthropometer P375 (Co. TRYSTOM, spol. s r.o./1993–2015 www.trystom.cz), with measurements taken to the nearest 0.1 cm.

Body weight was measured using a medical calibrated scale, TPLZ1T46CLNDBI300, with body weight recorded to the nearest 0.1 kg. Children were measured in a separate room, with the presence of teacher and examiner, in light t-shirts and shorts without shoes.

Skinfolds: subscapular (subsc), triceps (tric), suprailiac (suprail). Skinfolds measurement in all children was done by only one examinator. Each child was asked to remove their t-shirt behind the portable screen. Behind the screen, the skinfolds were measured. Values were dictated to the scorer who sat in front of the screen at a table and did not see the child. Skinfolds were measured with the Harpenden skinfold calliper, with an accuracy of 0.2 mm [17]. The latest available data on the thickness of triceps, subscapular and suprailiac for Czech children in age categories 9–9.99 and 10–10.99 were used as references [18]. 

An important assumption for the identification of normal-weight obese children was that those children will not differ by their BMI from normal-weight non obese peers but will have a significantly higher amount of body fat. In order to make sure that all children in the group of normal-weight obese individuals matched this requirement, we estimated in all three groups of children the percentage of body fat according to Slaughter’s equations [19].

For males with the sum of skinfolds less than 35 mm, the following equation was used:

*%BF* = 1.21 * *(tric + subsc)* − 0.008 * *(tric + subsc)*^2^ − 1.7

For females with the sum of skinfolds less than 35 mm, the following equation was used:

*%BF* = 1.33 * *(tric + subsc)* − 0.013 * *(tric + subsc)*^2^ − 2.5

For males with the sum of skinfolds higher than 35 mm, the following equation was used:

*%BF* = 0.783 * *(tric + subsc)* + 1.6

For females with the sum of skinfolds higher than 35 mm, the following equation was used:

*%BF* = 0.546 * *(tric + subsc)* + 9.7

#### 2.2.2. Physical Fitness Tests

(1) All tests were administrated during two physical education (PE) classes in the school gym in a fixed sequence. Before testing, children completed sufficient warm up, which had the same content in both PE classes. Each test was described, performed by a trained examiner and all instructions were provided. Participants came to the school gym and proceeded to take all tests in small four-member groups in a fixed sequence: (1) broad jump (dynamic strength (explosivity) of lower extremities); (2) 4 × 10 m running (speedy coordination); (3) sit ups for 60 s (endurance strength of trunk muscles and flexors of hips); (4) 20 m shuttle running (Leger test) (aerobic endurance). Details about each test are provided in Table 1.

Physical fitness was measured by selected fitness tests, which were validated and are fully standardized in a Czech environment [20]:

For detailed description of tests, see: UNIFITTEST (6–60) Test and Norms of Motor Performance and Physical Fitness in Youth and in Adults [20].

### 2.3. Selection of Final Sample in Relation to the Aim of Study

For selection of the final sample for the current study, we had to carry out the following three steps: (1) children categorization; (2) excluding regularly sporting children; (3) excluding children with different biology maturation status.

In the first step, based on anthropometry parameters we categorized children into three categories (1) normal-weight obese; (2) normal-weight non obese; (3) overweight and obese.

We used:

World health organization (https://www.who.int/growthref/who2007_bmi_for_age/en/) BMI reference with percentile cut-off points and Czech national references of the three skinfolds (over triceps, subscapular, suprailliac) children in age 9.01–10.99 [18].

The criteria for each defined group of children were as follows:

(1) Children with normal-weight obesity were those whose BMI (WHO reference chart) was in the 25th–75th percentile range along with average value from three skinfolds exceeding the 85th percentile of the Czech national reference. The narrower range of BMI for normal-weight obese was selected in order to avoid significant non-equality of BMI between normal-weight obese and normal-weight non obese, which is what happened, e.g., in [20]. Our aim was to select a population of normal-weight obese and normal-weight non obese that would be indistinguishable based on BMI.
(2) Normal weight non-obese individuals were those whose BMI (WHO reference chart) and average value from three skinfolds were in the 15th–84th percentile range of the Czech national reference.
(3) Children who were overweight and obese were those whose BMI (WHO reference chart) and average value from three skinfolds exceeded the 85th and 95th percentile of the Czech national reference, respectively.

Level of physical fitness is significantly influenced by regular participation in sport. Therefore, in the second step we investigated the proportion of children doing regular (at least once a week) organized team or individual sport activity. Since we found that none of the children who were obese or normal-weight obese participated in regular sport activities therefore, children in the normal-weight non-obese category (*n* = 103) and overweight category (*n* = 23) who regularly participated in sport were excluded from the analysis in this study. However, given the complexity and variation in sports participation and associated intensities, we were unable to account for this in our analysis.

In the third step of final sample selection, in order to ameliorate the impact of biological/maturation vs. chronological age discrepancies on body fat and physical fitness, maturity status was assessed. Since in selected age categories differences in biology maturation occurred, the Khamis Roche method for assessing the biological maturation status of children was used.

Age, height, and weight of the children and mid-parental height were used to predict adult height. To calculate mid-parental height, personal heights from both biological parents of each child were obtained. The current height of each child was then converted to a percentage of their predicted adult height, which is an indicator of maturity status.^35^ Further, we compared the percentage of predicted adult height for each child with sex-specific growth reference data for the Czech Republic [18] to derive an index of biological age (BA). Maturity status was estimated as BA minus CA (BA-CA). Participants were subsequently classified as on-time or average if the difference between BA and CA fell within ±1.0 year, advanced if the difference was >+1.0 year, and delayed if the difference was >−1.0 year.

In 27 children (boys = 11; girls = 16), maturity status significantly differs from chronological age, therefore these children were removed from the analysis. From 27 removed children, 18 were biologically advanced: 7 boys and 11 girls, and 9 participants were biologically late maturing: 5 boys and 4 girls.

The research sample finally consisted of 328 children, from 9.00 to 10.99 years old (boys = 158; girls = 170) (Table 2).

### 2.4. Statistical Analysis

A Shapiro–Wilk test initially determined that the data were not normally distributed (*p* < 0.001), following which, to account for skewness, we log transformed all raw data to normality before further analysis [21]. Differences between sexes were assessed using independent samples *t*-tests. In order to investigate between-group differences (normal-weight obese, normal-weight non obese, overweight and obese), an analysis of covariance (ANCOVA) was conducted, accounting for the unequal variance in the groups. We controlled initially for Sex and Maturation status; however, in instances where covariates were not significant, they were removed from the model in pursuit of the most parsimonious solution. Where appropriate, we employed Games–Howell post-hoc tests, which also account for unequal variance, to further explore sources of difference. We also performed a Welch ANOVA for sex*group (normal-weight obese m, normal-weight obese f, normal-weight non obese m, normal-weight non obese f, overweight and obese m, overweight and obese f), followed by Games–Howell post hoc tests. All data analysis was conducted using Statistical Package for the Social Sciences (SPSS) (IBM statistics, Version. 24, Armonk, NY, USA). Within the manuscript, we present the untransformed data for ease of interpretation. Statistical significance was accepted at *p* < 0.05.

## 3. Results

We compared the anthropometric profile of all defined categories; normal-weight obese, normal-weight non obese, and overweight and obese, considering sex. Since the age did not differ significantly across groups F (325, 2) = 2.8, *p* = 0.09, we were able to report the actual values. Post Hoc comparisons revealed that children who were normal-weight non obese (boys and girls) were significantly shorter, lighter and had a significantly lower BMI compared to peers who were overweight and obese. Children who were normal-weight obese were also significantly lighter and had a lower BMI compared to overweight and obese counterparts; however, only girls who were normal-weight obese were significantly shorter than girls who were overweight and obese. Further, children who were normal-weight non obese and normal-weight obese did not differ in their BMI. However, normal-weight obese boys and girls had a significantly higher percentage of body fat compared to normal-weight non obese peers. In addition, children who are overweight and obese had the highest values of body fat, but they were not significantly higher than normal-weight obese peers (Table 3 and Table 4), whilst overall, and in each weight category, females had significantly higher BF% than males (all: *p* < 0.001).

The post hoc analysis of PF tests showed that normal-weight obese children achieved significantly worse performances in tests of strength and explosiveness of lower limbs (broad jump), speed coordination (4 × 10 m shuttle run) and endurance (Leger test) compared to normal-weight non obese peers. On the other hand, these normal-weight obese children did not significantly differ in the aforementioned fitness tests from overweight and obese counterparts who achieved on average the worst performances (Table 3). For detailed comparison of results in post hoc analysis, please see Appendix A.

**Figure 3:**
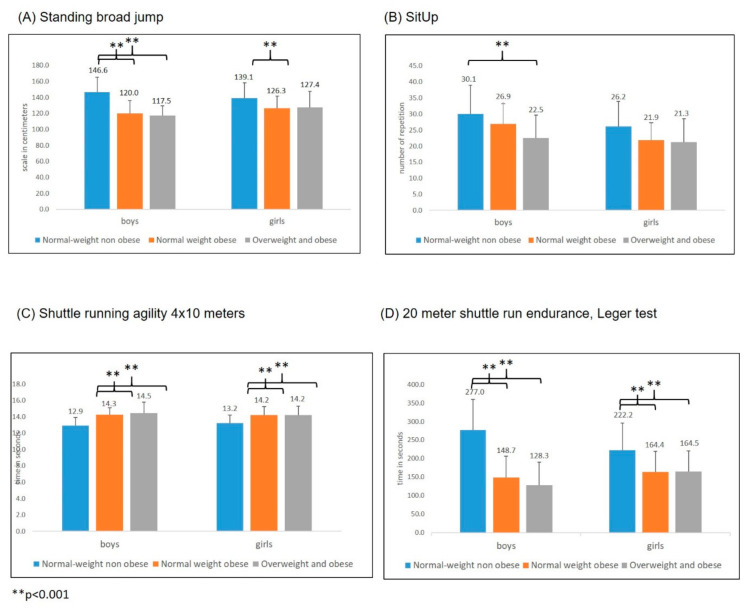
Differences in lower limb explosivity, strength endurance of trunk muscles, running agility and endurace performances between Normal-weight non obese, Normal-weight obese and Overweight and obese groups considering sex.

Finally, when we looked into the results of boys and girls separately, we revealed that normal-weight obese and overweight and obese boys displayed higher evidence for physical fitness deficits in strength and explosiveness of lower limbs, speed coordination and endurance compared to normal-weight non obese peers (Figure 3).

The largest deficit of normal-weight obese boys was found in the Leger cardio-respiratory test where normal-weight obese boys achieved on average ~128 s (more than 2 min) less time compared to normal-weight non obese counterparts. This difference equates to normal-weight obese boys achieving only 54% of the average cardio-respiratory performance of normal-weight non obese boys.

## 4. Discussion

The aim of the current study was to investigate the physical fitness of children with normal-weight obesity, in comparison to normal-weight non obese and overweight and obese counterparts, respectively. The principal, and novel, finding of this study is that normal-weight obese children have a significantly lower level of muscle strength explosiveness (broad jump), and significantly lower level of agility (4 × 10 m test), where high speed coordination demands on movement pattern are required, compared to normal weight non-obese peers. Additionally, normal-weight obese children were characterised by equally poor endurance, strength of lower extremities, and agility as children with overweight and obesity. Such information conceivably corresponds with the finding of [13], where normal-weight obese pre-school children had significantly poorer fundamental motor skills (FMS). In particular, balance and postural stability, as a tenet of FMS, were shown to be highly correlated with the development of motor coordination [22].

A large body of evidence has demonstrated that a significant link exists between physical fitness and health status in children and adolescents [23]. In a review [24], it was concluded that both cardio-respiratory and muscular fitness are related with cardiovascular disease risk factors, and that muscular fitness and speed/agility seem to elicit a positive effect on bone health. An important extension of the aforementioned relationships is that physical fitness level is also positively linked to long term health status [25]. Thus, children with better PF in childhood have a lower risk of health-related diseases (cardiovascular, metabolic) in adulthood [26]. Moreover, it has been found that physical fitness is strongly associated with physical activity regimes [27] and that a transfer exists between physical activity in childhood and adulthood, with consequential health implications [28].

Importantly, the findings of the present study highlight the importance of examining physical fitness in children, because children who are normal-weight obese are indistinguishable from normal weight non-obese counterparts through standard surveillance protocols. Indeed, Lang et al. [29] asserted that cardiorespiratory fitness, and related fitness parameters, are important indicators of current and future health among school-aged children and youth, and, importantly, are independent of physical activity levels; such information could be used to compare across regions to help identify healthy and unhealthy populations, allowing policy-makers and educationalists to implement appropriate actions. Moreover, repeated measurements in the same population could yield insight into temporal health trends, which may be useful in monitoring the impact of implemented policies [30].

Currently, there are few examples of national physical fitness surveillance; however, a laudable example of how national fitness surveillance can benefit children and youth, comes from Slovenia. Indeed, Slovenia collects national physical fitness data for all children (6–19 y), through an initiative called “SLOfit” [30], and has been obligatory for more than three decades. Following this approach, researchers, educators, and policymakers identified a receding trend in fitness between 1990 and 2010; accordingly, Slovenia implemented a national health-promoting intervention, coinciding with the 2010/2011 school year. Consequently, the physical fitness levels of Slovenian children have notably improved. Indeed, the recent Global Physical Activity Report card, representing six continents [31], showcased that Slovenia was ranked number one in overall national physical activity levels among youth [31], highlighting the importance of physical fitness surveillance.

Further consideration should be given to sex-related differences; indeed, when we used the sex of participants as a covariate, the differences in physical fitness tests between normal-weight obese and normal-weight non obese children were much larger in magnitude in males. However, when we included age and maturation as covariates, they were non-significant in all cases, which may be due to the small variance in age and maturation status in the present sample. Clearly, however, larger samples, across a wider age range should be conducted to better discern the influence of age. It is interesting that in three tests of muscle strength, explosiveness of the lower limbs, agility and cardio-respiratory capacity, normal-weight obese boys achieved scores lower than that of normal-weight obese girls. This further evidence of the poor physical fitness level normal-weight obese boys shows that normal-weight obesity might have more serious outcomes in the male population, and thus warrants detailed, further examination. Indeed, we advocate that further work be conducted to better discern the role of sex in normal weight obese children and possible different health risks between normal-weight obese males and females. Normal weight obesity appears to be time stable phenomena from childhood to early adulthood; we therefore suggest that future research investigate, longitudinally, PA regimes, nutritional habits, and cognitive development of normal-weight obese children. Weak performance in PF tests can be explained, at least in part, by the comparatively lower lean mass of normal-weight obese children. Indeed, it was reported by [32] that poor PF performance was partly (up to 25%) attributable to low skeletal muscle mass of normal-weight obese individuals. Further, we suggest that normal-weight obese individuals have a generally lower level of motor competence, which was supported in [13]. In line with the conceptual model of [3], it is apparent that physical activity may be directly influencing, not only lean mass development, but also movement experience, which is essential for successful completion of any movement task). In addition, it should be noted that previous studies, regardless of which method they employed to identify normal-weight obese individuals, have highlighted that normal-weight obese populations were always, according to self-reported questionnaires, less physically active [23,33,34].

In summary, normal-weight obese children have a correspondingly low physical fitness level to their overweight and obese peers, which likely negatively affects their health status regarding cardio-respiratory, metabolic, and skeletal and muscle development through the life course. Moreover, the current study implies that normal weight obesity could be sex dependent, even in childhood, regarding the degree of deficits, suggesting that discrepancies between normal-weight obese and normal-weight non obese boys were of a much greater magnitude than in girls.

### Strengths and Limitations

The principal strength of the present study is the homogeneous research sample, consisting of a narrow age range, within the period of stable motor and growth development, and although this can reduce generalizability, the precise and specific sample permits accurate insight into this age group of children. A further strength of this study is that we controlled leisure time activities so that performances in PF tests were not confounded by children’s participation in sport or organized physical activities. On the other hand, unbalanced samples in each category, and the fact that children were only selected from a capital city, represent limitations of the present study, where we are unable to generalize our results to wider demographics and ages. Accordingly, when we attempted to use age and maturation as covariates, they were not significant, thus highlighting the need for wider age ranges to further this work. Additionally, seasonality was not considered in the data acquisition or analysis, which might conceivably have some impact. A further limitation is that we were not able to quantify, or account for, the pubertal stage of the participants. Indeed, the authors must concede that pubertal stage could have influenced the results, particularly regarding the comparability of boys vs. girls; thus, we suggest that further work be undertaken in this regard. Finally, the present study was cross-sectional in design, and therefore, causality cannot be discerned.

## 5. Conclusions

Normal-weight obese children showed comparable deficits in both cardio-respiratory and muscular fitness as their overweight and obese peers, compared to children who are normal-weight non obese. Moreover, the current study supports previous suggestions that normal weight obesity could be sex dependent. Further, because children who are normal-weight obese are indistinguishable from normal weight non-obese counterparts through standard surveillance protocols, identification, surveillance, and designing health enhancing interventions is problematic, and thus, a concerted effort must be made to discern physical fitness levels throughout childhood and adolescence, particularly in school-settings, using nationally representative surveillance methods.

## Figures and Tables

**Figure 2:**
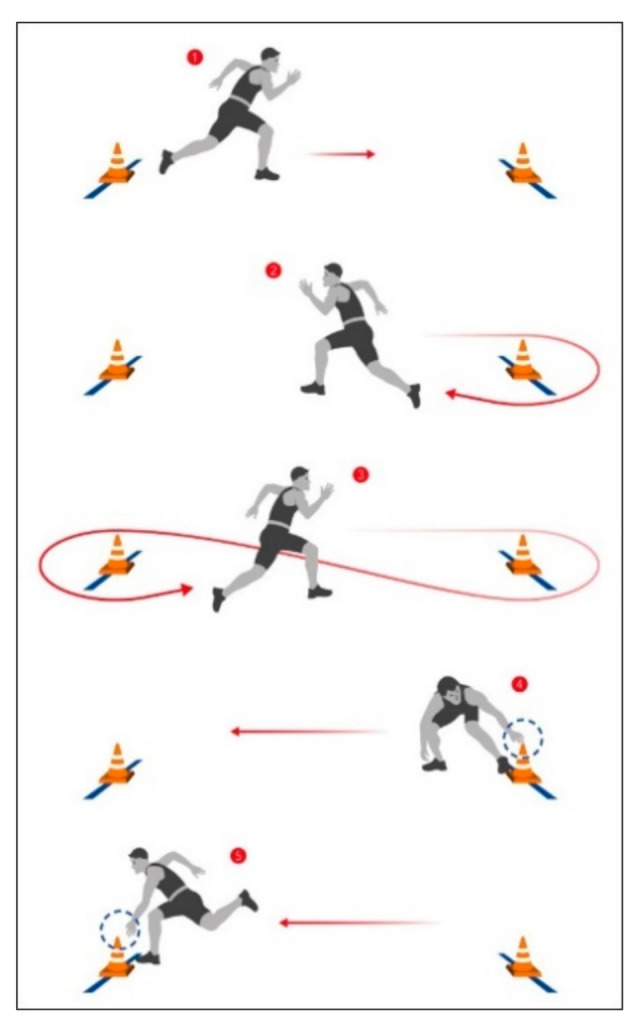
Four ×10 m shuttle running.

**Table 1:** Description of physical fitness tests.

Test	Description
Broad jump:	Looks forward and jumps as far as possible. The test is scored in centimetres. There are three trials and the best attempt is scored.
4 × 10 m shuttle running:	Four × 10-m shuttle running, consecutively. The test is scored in seconds and tenths of seconds. There are two trials and the better time is scored; see Figure 2 below
SitUps for 60 s:	The child lies on their back on a small (3 cm tall) soft mat, arms bent, hands behind the neck. The knees are bent at 90°. Feet are fixed to the ground by the examiner. The examiner counts the number of properly repeated cycles during 60 s.
20 m shuttle run endurance test (Leger test):	The child repeatedly runs back and forth between two lines that are set 20 m apart according to a sound signal that progressively increases the speed. At each sound signal, the child must reach the line. The test ends when the child fails to reach the end lines prior to the beep on two successive occasions. Number of stages and time are scored.

**Table 2:** Frequency distribution of children from *n* = 328.

Category	Boys	Girls	Boys & Girls
Normal-weight obese (NWO)	14	30	44
Normal-weight non obese (NWNO)	116	121	237
Overweight and obese (OwOb)	28	19	47
Age (y)	9.9 (0.5)	9.8 (0.5)	9.8 (0.5)

Note: Age is presented in years (standard deviation).

**Table 3:** Anthropometry profile of children in three defined categories considering sex.

**BOYS**	**NWNO (n = 124)**	**NWO (n = 15)**	**Ow & Ob (n = 31)**	***p*-Value**
Height (cm)	142.5 ± 4.7 ^(a)^	143.4 ± 4.1	146.7 ± 4 ^(a)^	<0.001
Weight (kg)	34.7 ± 3.2	34.1 ± 4.1	51.3 ± 5.9 ^(b)^	<0.001
BMI^1^	16.7 ± 1.3	16.6 ± 1.5	23.8 ± 2 ^(b)^	<0.001
Body fat%	16.2 ± 3.3 ^(c)^	28.4 ± 3.4	31 ± 5.2	<0.001
**GIRLS**	**NWNO (n = 132)**	**NWO (n = 32)**	**Ow & Ob (n = 21)**	***p*-Value**
Height (cm) ^D^	140.8 ± 4.9	142.9 ± 4.2	147.4 ± 3.4 ^(b)^	<0.001
Weight (kg) ^D^	32.1 ± 3.9	34.1 ± 3.4	51.1 ± 7 ^(b)^	<0.001
BMI^1^	16.2 ± 1.3	16.7 ± 1.1	23.4 ± 2.4 ^(b)^	<0.001
Body fat%	18.5 ± 4 ^(c)^	30.4 ± 3.8	33.9 ± 5.1	<0.001

Games–Howell Post Hoc Multiple Comparisons: ^D^ data are presented as mean ± SD. Letters ^(a–c)^, marked which group or groups significantly differ on level *p* < 0.001. Difference between NWNO and Ow & Ob groups. (a) Difference between Ow & Ob and NWO and between Ow & Ob and NWNO groups; no significant difference between NWO and NWNO. (b) Difference between NWNO and NWO and Ow & Ob groups; no significant difference between NWO and Ow & Ob. Abbreviations: NWNO = normal-weight non obese; NWO = normal-weight obese; Ow & Ob = overweight and obese; BMI = body mass index.

**Table 4:** Main effect in physical fitness between the three defined groups of children.

	NWNO (n = 256)	NWO (n = 47)	Ow & Ob (n = 52)	F-Ratio	*p*-Value
Broad jump cm ^D^	142.7 ± 19.3 ^(c)^	124.3 ± 15.7	121.7 ± 16.6	41.5	<0.001
SitUp No. repetition ^D^	28.1 ± 7.5 ^(c)^	23.5 ± 6.1	22 ± 7.2	16.3	<0.001
4 × 10 m s ^D^	13.1 ± 1 ^(c)^	14.2 ± 0.9	14.4 ± 1.2	51	<0.001
Leger test ^D^	248.7 ± 82.5 ^(c)^	159.4 ± 55	143.6 ± 62	69.7	<0.001

^D^ data are presented as mean ± SD. ^(c)^ Difference between NWNO and NWO and Ow & Ob groups, no significant difference between NWO and Ow & Ob. Abbreviations: NWNO = normal-weight non obese; NWO = normal-weight obese; Ow & Ob = overweight and obese.

## Appendix A

**Table A1:** Post hoc analysis and certain differences in physical fitness tests between the three groups of children.

BROAD JUMP	NWNO (n = 256)	NWO (n = 47)	Ow & Ob (n = 52)
NWNO (*n* = 256)	0		
NWO (*n* = 47)	−18.4 cm **	0	
OW&OB (*n* = 53)	−21 cm **	−2.6 cm	0
SITUP			
NWNO (*n* = 256)	0		
NWO (*n* = 47)	−4.6 rep **	0	
OW&OB (*n* = 53)	−6.1 rep **	−1.5 rep	0
4 × 10 METER			
NWNO (*n* = 256)	0		
NWO (*n* = 47)	+1.1 s **	0	
OW&OB (*n* = 53)	+1.3 s **	+0.2 s	0
LEGER TEST			
NWNO (*n* = 256)	0		
NWO (*n* = 47)	−1:29 min **	0	
OW&OB (*n* = 53)	−1:45 min **	−0:16 min	0

** *p* < 0.001. Abbreviations: rep = number of repetitions; NWNO = normal weight non obese; NOW = normal-weight obese obese; Ow & Ob = overweight and obese.

## References

[1] Simons-Morton B.G., Parcel G.S., O’Hara N.M., Blair S.N., Pate R.R. (1988). Health-related physical fitness in childhood: Status and recommendations. Ann. Rev. Public Health.

[2] DuBose K.D., Eisenmann J.C., Donnelly J.E. (2007). Aerobic fitness attenuates the metabolic syndrome score in normal-weight, at-risk-for-overweight, and overweight children. Pediatrics.

[3] Stodden D.F., Goodway J.D., Langendorfer S.J., Roberton M.A., Rudisill M.E., Garcia C., Garcia L.E. (2008). A developmental perspective on the role of motor skill competence in physical activity: An emergent relationship. Quest.

[4] Torrijos-Niño C., Martínez-Vizcaíno V., Pardo-Guijarro M.J., García-Prieto J.C., Arias-Palencia N.M., Sánchez-López M. (2014). Physical fitness, obesity, and academic achievement in schoolchildren. J. Pediatr..

[5] De Lorenzo A., Del Gobbo V., Premrov M.G., Bigioni M., Galvano F., Di Renzo L. (2007). Normal-weight obese syndrome: Early inflammation?. Am. J. Clin. Nutr..

[6] Jean N., Somers V.K., Sochor O., Medina-Inojosa J., Llano E.M., Lopez-Jimenez F. (2014). Normal-weight obesity: Implications for cardiovascular health. Curr. Atheroscler. Rep..

[7] Musalek M., Pařízková J., Godina E., Bondareva E., Kokštejn J., Jírovec J., Vokounová Š. (2018). Poor skeletal robustness on lower extremities and weak lean mass development on upper arm and calf: Normal weight obesity in middle-school-aged children (9 to 12). Front. Pediatr..

[8] Frost H.M. (2003). Bone’s mechanostat: A 2003 update. Anat. Rec. Part A Discov. Mol. Cell. Evol. Biol. Off. Publ. Am. Assoc. Anat..

[9] Olafsdottir A.S., Torfadottir J.E., Arngrimsson S.A. (2016). Health behavior and metabolic risk factors associated with normal weight obesity in adolescents. PLoS ONE.

[10] Ruderman N., Chisholm D., Pi-Sunyer X., Schneider S. (1998). The metabolically obese, normal-weight individual revisited. Diabetes.

[11] Zhang M., Schumann M., Huang T., Törmäkangas T., Cheng S. (2018). Normal weight obesity and physical fitness in Chinese university students: An overlooked association. BMC Public Health.

[12] Malina R.M., Bouchard C., Bar-Or O. (2004). Growth, Maturation, and Physical Activity.

[13] Haga M. (2008). The relationship between physical fitness and motor competence in children. Child Care Health Dev..

[14] Musalek M., Kokstejn J., Papez P., Scheffler C., Mumm R., Czernitzki A.F., Koziel S. (2017). Impact of normal weight obesity on fundamental motor skills in pre-school children aged 3 to 6 years. Anthropol. Anz..

[15] Magnussen C.G., Schmidt M.D., Dwyer T., Venn A. (2012). Muscular fitness and clustered cardiovascular disease risk in Australian youth. Eur. J. Appl. Physiol..

[16] Vignerová J., Riedlová J., Bláha P., Kobzová J., Krejčovský L., Brabec M., Hrušková M. (2006). 6. Celostátní antropologický výzkum dětí a mládeže 2001 Česká republika. Souhrnné Výsledky, 6th Nation-Wide Anthropological Survey of Children and Adolescents 2001 Czech Republic.

[17] Di Renzo L., Sarlo F., Petramala L., Iacopino L., Monteleone G., Colica C., De Lorenzo A. (2013). Association between−308 G/A TNF-α polymorphism and appendicular skeletal muscle mass index as a marker of sarcopenia in Normal-weightly obese syndrome. Dis. Markers.

[18] Lohman T.G., Roche A.F., Martorell R. (1988). Anthropometric Standardization Reference Manual.

[19] Carter J.L., Heath B.H. (1990). Somatotyping: Development and Applications.

[20] Slaughter M.H., Lohman T.G., Boileau R., Horswill C.A., Stillman R.J., Van Loan M.D., Bemben D.A. (1988). Skinfold equations for estimation of body fatness in children and youth. Hum. Biol..

[21] Chytráčkovč J., Měkota K. (2002). Unifittest (6–60): Příručka pro Manuální a počÍtačové Hodnocení Základní Motorické Výkonnosti a Vybraných Charakteristik Tĕlesné Stavby Mládeže a Dospĕlých v České Republice. The Test Manual for Computer Assessing of Physical Fitness and Selected Characteristic of Body Status in Youth and Adults in Czech Republic.

[22] Sedgewick P. (2012). Log transformation of data. BMJ.

[23] Feldman A.G. (1986). Once more on the equilibrium-point hypothesis (λ model) for motor control. J. Mot. Behav..

[24] Madeira F.B., Silva A.A., Veloso H.F., Goldani M.Z., Kac G., Cardoso V.C., Bettiol H., Barbieri M.A. (2013). Normal weight obesity is associated with metabolic syndrome and insulin resistance in young adults from a middle-income country. PLoS ONE.

[25] Smith J.J., Eather N., Morgan P.J., Plotnikoff R.C., Faigenbaum A.D., Lubans D.R. (2014). The health benefits of muscular fitness for children and adolescents: A systematic review and meta-analysis. Sports Med..

[26] Ortega F.B., Ruiz J.R., Castillo M.J., Sjöström M. (2008). Physical fitness in childhood and adolescence: A powerful marker of health. Int. J. Obes..

[27] Malina R.M. (2001). Physical activity and fitness: Pathways from childhood to adulthood. Am. J. Hum. Biol. Off. J. Hum. Biol. Assoc..

[28] Donnelly J.E., Hillman C.H., Castelli D., Etnier J.L., Lee S., Tomporowski P., Lambourne K., Szabo-Reed A.N. (2016). Physical activity, fitness, cognitive function, and academic achievement in children: A systematic review. Med. Sci. Sports Exerc..

[29] Telama R., Yang X., Viikari J., Välimäki I., Wanne O., Raitakari O. (2005). Physical activity from childhood to adulthood: A 21-year tracking study. Am. J. Prev. Med..

[30] Lang J., Tomkinson G., Janssen I., Ruiz J., Ortega F., Leger L., Tremblay M. (2018). Making a Case for Cardiorespiratory Fitness Surveillance Among Children and Youth. Exerc. Sport Sci. Rev..

[31] Strel J. (2013). Analysis of the Program Healthy Lifestyle for the Years 2010/11 and 2011/12 [Article in Slovenian].

[32] Tremblay M.S., Barnes J.D., Gonzalez S.A., Katzmarzyk P.T., Onywera V.O., Reilly J.J., Tomkinson G.R. (2016). Global Matrix 2.0 Research Team. Global Matrix 2.0: Report card grades on the physical activity of children and youth comparing 38 countries. J. Phys. Act. Health.

[33] Khamis H.J., Roche A.F. (1994). Predicting adult stature without using skeletal age: The Khamis-Roche method. Pediatrics.

[34] Roche A.F., Tyleshevski F., Rogers E. (1983). Non-invasive measurements of physical maturity in children. Res. Q. Exerc. Sport.

